# May predictors of difficulty in thyroid surgery increase the incidence of complications? Prospective study with the proposal of a preoperative score

**DOI:** 10.1186/s12893-018-0447-7

**Published:** 2019-04-24

**Authors:** Valerio D’Orazi, Andrea Sacconi, Silvia Trombetta, Menelaos Karpathiotakis, Daniele Pichelli, Enrico Di Lorenzo, Alice Ortensi, Paolo Urciuoli, Marco Biffoni, Andrea Ortensi

**Affiliations:** 1 Department of Surgical Sciences, “Sapienza” University, Viale Regina Elena 324, 00161, Rome, Italy; 2 Department of General Microsurgery and Hand Surgery, “Fabia Mater” Hospital, Rome, Italy; 3 Translational Oncogenomics Unit, Molecular Medicine Area, “Regina Elena” National Cancer Institute, Rome, Italy; 4 Department of General Microsurgery and Hand, Surgery Section of phoniatrics and speech therapy, “Fabia Mater” Hospital, Rome, Italy; 5 Department of General Microsurgery and Hand Surgery, Section of physiotherapy, “Fabia Mater” Hospital, Rome, Italy; 6 Chief of Department of General Microsurgery and Hand Surgery, “Fabia Mater” Hospital, Rome, Italy

**Keywords:** Thyroid surgery, Difficult thyroidectomy, Predictive factors, Preoperative difficulty score

## Abstract

**Background:**

Although thyroidectomy is one of the most common surgical procedures performed worldwide, some permanent complications, despite the considerably reducing incidence, may affect dramatically the patients quality of life. The purpose of this study is to evaluate whether factors identified preoperatively and expressed in a score could be predictors of major surgical difficulty during total thyroidectomy and influence the incidence of complications.

**Methods:**

A total of 164 patients who underwent total thyroidectomy were examined. For each patient we calculated a preoperative score, including seven parameters, which we evaluated to be predictors of difficulty in thyroid surgery, that is, sex, body mass index (BMI), neck length, neck extension, thyroid gland volume, thyroiditis, and increased parenchymal vascularization. The overall score was also compared with peri- and post-operative factors describing objectively the difficulty in thyroid surgery. These factors are the duration of the operation, the length of hospitalization, the incidence of complications such as hemorrhage, hypoparathyroidism, and recurrent laryngeal nerve injuries.

**Results:**

There was no statistically significant association between our score and either the percentage of postoperative complications or the length of hospitalization. The operative time was the only variable remarkably associated with the score value (*p* < 0.00001). Comparing the duration of the operation with each of the preoperative predictive factors, we found that none of the factors reached the value of statistical significance, but a close association could be noted with the thyroid volume and the BMI.

**Conclusions:**

In our study, predictors of difficulty in thyroidectomy did not affect morbidity rates, as suggested by previous studies, but only operative times, which were significantly increased in patients with higher score. Although our results have limited statistical significance, they allow us to confirm the fundamental role of a systematic use of optical magnification and microsurgical technique in thyroidectomy. Further studies, with a larger cohort of patients, are needed to validate our results and to formulate a universally accepted predictive score of difficulty in thyroidectomy preoperatively.

## Background

Thyroidectomy is one of the most common surgical procedures performed worldwide. Over 34,000 operations were performed during 2016 in Italy and the incidence of thyroid diseases that require total thyroidectomy is constantly increasing [1].

Historically, mortality in thyroid surgery drastically reduced from about 50% in the dawn of the last century to a negligible rate nowadays, thanks to the improvement of surgical techniques, the introduction of technological innovations, and the improved knowledge of pathophysiology. Therefore, the incidence of perioperative complications is around 1–2% [2–4]. However, permanent complications, such as vocal cord palsy and hypoparathyroidism, can dramatically reduce the quality of life of patients and have always represented a topic of intense clinical interest and research for most neck surgeons.

The literature shows a strong evidence that the incidence of perioperative complications correlates to factors such as thyroid disease, specific characteristics of each patient or the thyroid gland, surgeon’s experience and surgical technique, even with the use of optical magnification tools [5–11].

The purpose of our study was to evaluate whether preoperative factors, grouped into a score and predictive of major surgical difficulty in thyroidectomy, might influence the incidence of complications, in order to improve patient’s management and optimize the planning of the operating theater.

## Methods

For this prospective study, a database of 195 consecutive patients who underwent total thyroidectomy from 01/01/2015 to 30/06/2017 in our center was reviewed. All patients obtained written informed consent. Operations were performed by two experienced surgeons of the microsurgery team. Exclusion criteria were lobectomy, reoperative thyroidectomy, concomitant parathyroidectomy and lymph node dissection for malignancy. Therefore, a cohort of 164 patients who had total thyroidectomy was examined. The minimum follow-up period was 6 months.

For each patient, during the preoperative assessment, a score was calculated including the following seven parameters, which we evaluated to be predictors of difficulty in thyroid surgery: sex, body mass index (BMI; Kg/m^2^), neck length (cm), neck extension, thyroid gland volume (ml), laboratory evidence of thyroiditis (high anti-Tg Ab levels), and Doppler-ultrasound evidence of increased parenchymal vascularization.

Routinely, all patients undergoing total thyroidectomy at our institution have a clinical assessment including thyroid function laboratory tests (dosage of free levels of T3 and T4, TSH, calcitonin, thyroglobulin-Tg, Ab anti-Tg, Ab anti-TPO), pre- and postoperative fibrolaringoscopy and thyroid Doppler ultrasonography. The radiologists provide us the value of the thyroid volume and the pattern of parenchymal vascularization.

The length of the neck was estimated by measuring the distance in cm between the jugular notch and the cricoid cartilage, considering a length of 6 cm as the lower threshold. The extensibility of the neck, evaluated preoperatively by the anesthesiologists, was distinguished in normal or reduced, regardless of the pathology (arthritis, cervical hernias, previous traumas, etc.).

Our surgical experience suggests that not all the risk factors identified preoperatively affect the difficulty of the surgical procedure equally. Thus, we decided to assign a different value in each of these parameters based on the empirical relevance they could have regarding the grade of surgical difficulty. Once established and calculated the individual value for each of the parameters, we formulated the overall score, with a range of values ​​from 0 to 15.

To stratify the obtained results, we identified three groups: patients with a low score (0–3), medium score (4–5), and high score (≥ 6) (Table 1).

**Table 1:** Preoperative parameters and score calculation

Sex	Female	0
	Male	1
BMI (kg/m^2^)	< 30	0
	≥ 30	1
Neck length (cm)	≥ 6 cm	0
	< 6 cm	2
Neck extensibility (cm)	Normal	0
	Reduced	2
Thyroid volume (ml)	≤ 80 ml	0
	> 80 ml	3
Hypervascularization	No	0
	Yes	3
Thyroiditis	No	0
	Yes	3
Score level	*Low*	*0–3*
	*Medium*	*4–5*
	*High*	*6–15*

The overall score was also compared with peri- and post-operative factors describing objectively the difficulty encountered during surgery. These factors are the duration of the operation, the length of hospitalization, the incidence of complications such as hemorrhage, hypoparathyroidism, and recurrent laryngeal nerve (RLN) injuries, either transient or permanent. The duration of the operation was obtained from the electronic operative register. Hypoparathyroidism was defined as the need for supplementary therapy with calcium and vitamin D. RLN injury was documented by postoperative fibrolaringoscopy, which is usually performed about 20 days after surgery. Complications were defined as transient in the event of complete resolution within 6 months from surgery, otherwise they were considered permanent.

The aim of our study was to compare the score of the population with all the peri- and post-operative parameters above mentioned, in order to understand whether the suggested model could be useful and reliable to predict the difficulty of thyroidectomy.

Statistical significance of the association between score level and categorical variables was assessed by using a Chi-square test.

### Surgical technique

All patients included in the study underwent total extracapsular thyroidectomy, in microsurgical technique using 4,5X loupes magnification by two experienced neck surgeons. We performed a Kocher incision of about 6 cm. After dissection of the superior and inferior subplatysmal flap, the strap muscles were divided longitudinally in the midline and the thyroid lobe was exposed and medially dislocated, once the middle thyroid vein was divided. We proceeded with the dissection of the superior pole, by separated ligation and division of the two branches of the superior thyroid artery, identifying and preserving the upper parathyroid and the motor branch of the superior laryngeal nerve. The ipsilateral RLN was identified with digital technique, traced along its entire cervical course, up to the entrance in the larynx. The distal branches of the inferior thyroid artery were divided, identifying and preserving the inferior parathyroid gland with its vascular peduncle. The same procedure was performed for the contralateral lobe, completing the thyroidectomy by removing the pyramidal lobe. Hemostasis was performed using bipolar electrocautery and microsurgical ligatures. The cervicotomy was closed after placement of two suction drains. In our Department, the use of ultrasound and radiofrequency devices for coagulation and tissue dissection is well established.

## Results

A population of 164 patients who underwent total extracapsular thyroidectomy in microsurgical technique was included in the study. Patients’ demographics, clinical and preoperative features and score values are summarized in Table 2. Post-operative data and outcomes are summarized in Table 3. Table 4 shows the statistical correlation through Chi-square test among the score level and the postoperative variables. We considered statistically significant values ​​of *p* < 0.001. Complications were considered as a whole and individually. No permanent hypoparathyroidism or RLN injury have been reported. About the length of hospitalization, we identified two groups of patients, accepting 2 days as the normal stay. Regarding the duration of surgery, patients were divided into two groups according to the median of 95 min.

**Table 2:** Demographic and pathophysiological characteristics of the population and overall score

Period of study Jan 2015 - July 2017	Number	Percentage (%)
Patients	164	
Sex		
▪ Female	135	82.3
▪ Male	29	17.7
Age		
▪ Range	21–79	
▪ Average	54.0	
BMI (Kg/m^2^)		
▪ < 30	131	79.9
▪ ≥ 30	33	20.1
▪ Average	26.1	
Neck length (cm)		
▪ ≥ 6 cm	150	91.5
▪ < 6 cm	14	8.5
Neck extensibility (cm)		
▪ Normal	144	87.8
▪ Reduced	20	12.2
Diagnosis		
▪ Goiter	87	53.0
▪ Hyperthyroidism	17	10.4
▪ Tir3A	10	6.1
▪ Tir3B	30	18.3
▪ Tir4	14	8.5
▪ Tir5	6	3.7
Thyroid volume (ml)		
▪ ≤ 80 ml	130	79.3
▪ > 80 ml	34	20.7
Hypervascularization		
▪ No	116	70.7
▪ Yes	48	29.3
Thyroiditis		
▪ No	111	67.7
▪ Yes	53	32.3
Overall score level		
▪ Low	115	70.1
▪ Medium	19	11.6
▪ High	30	18.3

**Table 3:** Peri- and postoperative parameters and surgical outcomes

Histology	Number	Percentage (%)
▪ Hyperplasia	60	36.6
▪ Thyroiditis	3	1.8
▪ Multinodular goiter	8	4.9
▪ Follicular adenoma	15	9.1
▪ FT-UMP^a^	1	0.6
▪ Malignancy:	77	46.9
Papillary ca n.o.s.	31	40.2
Papillary ca follicular variant	38	49.3
Papillary ca oncocitary variant	2	2.6
Papillary ca sclerosing variant	2	2.6
Follicular ca	2	2.6
Medullary ca	2	2.6
Duration of the operation (min)		
▪ Average	104.4	
▪ Median	95	
▪ Range	50–250	
Length of hospitalization (d)		
▪ Average	2.46	
▪ 2 days	108	65.9
▪ > 2 days	56	34.1
Complications		
▪ Hemorrhage	3	1.8
▪ Hypoparathyroidism (transient)	28	17.1
▪ Hypoparathyroidism (permanent)	0	/
▪ Recurrent laryngeal nerve lesion (transient)	1	0.6
▪ Recurrent laryngeal nerve lesion (permanent)	0	/

^a^ *FT-UMP* follicular tumor of uncertain malignant potential

**Table 4:** Correlations among score level and categorical perioperative variables (*p* < 0.001)

	Low risk (115 pts)	Medium risk (19 pts)	High risk (30 pts)	*p* value
Complications				0.52
▪ No	92	17	23	
▪ Yes	23	2	7	
*Hemorrhage*	*1*	*0*	*2*	
*Hypoparathyroidism*	*21*	*2*	*5*	
*RLN lesion*	*1*	*0*	*0*	
Length of hospitalization				0.85
▪ 2 days	75	12	21	
▪ > 2 days	40	7	9	
Duration of the operation				0.00001
▪ ≤ 95 min	72	6	5	
▪ > 95 min	43	13	25	
	Low risk (115 pts)		Medium/high risk (49 pts)	
Duration of the operation				0.000002
▪ ≤ 95 min	72		11	
▪ > 95 min	43		38	

The results showed no statistical significant association between our score and either the percentage of postoperative complications or the length of hospitalization. The operative time was the only variable remarkably associated with the score value (*p* < 0.00001). The association was even more significant if the groups of medium and high score level were combined (*p* < 0.000002).

Hence, we compared the duration of the operation with each of the preoperative predictors used for the score calculation to evaluate if any of these was more responsible for the correlation (Table 5). None of the factors reached the value of statistical significance, however a close association could be noted with the thyroid volume and the BMI (*p* = 0.0016 and 0.009 respectively).

**Table 5:** Correlation between duration of the operation and each of the variables used for score calculation

	Operative times ≤ 95 min (83 pts)	Operative times > 95 min (81 pts)	*p* value
Sex			0.055
▪ Female	73	62	
▪ Male	10	19	
BMI (Kg/m^2^)			0.009
▪ < 30	73	58	
▪ ≥ 30	10	23	
Neck length (cm)			0.96
▪ ≥ 6 cm	76	74	
▪ < 6 cm	7	7	
Neck extensibility			0.14
▪ Normal	76	68	
▪ Reduced	7	13	
Thyroid volume			0.0016
▪ ≤ 80 ml	74	56	
▪ > 80 ml	9	25	
Hypervascularization			0.012
▪ No	66	50	
▪ Yes	17	31	
Thyroiditis			0.95
▪ No	56	55	
▪ Yes	27	26	

## Discussion

Throughout the literature is possible to find different examples of scores and scales proposed to evaluate preoperatively the difficulty of the surgical procedure and based on specific risk factors associated with the type of the operation (e.g. nephrectomy etc.) [12–16].

However, about thyroid surgery, there are only few results for scores or scales predictive of surgical difficulty, which are defined only intra- or postoperatively [5, 17–20]. To our knowledge, there is no preoperative score able to evaluate predictors of difficulty in total thyroidectomy and detect patients with higher risk of complications.

In this study we identified some preoperative predictors of major surgical difficulty and consequently of higher risk of complications. The parameters analyzed were sex, BMI, length and extensibility of the neck, thyroid volume, hypervascularization, and thyroiditis.

For each factor we assigned a value from 0 to 3; the sum of each category determined the final score. According with this score, we divided the study population into three groups: high, medium and low level of surgical risk. Subsequently, each group was correlated with the incidence of complications (hemorrhage, permanent or transient RLN injury, and permanent or transient hypoparathyroidism), with the duration of the operation and the length of hospitalization.

In our opinion, the predictive factors represent those characteristics of the patient or of the thyroid disease that can influence the difficulty of the surgery. The choice of these parameters was made in accordance with our surgical experience and the existing scores.

For example, neck surgeons agree that certain thyroid disorders (thyroiditis, hyperthyroidism) are associated with pathophysiological features of the gland that can make the dissection quite difficult [21–23], as well as some more aggressive thyroid tumors [24–26]. There is even the possibility that some thyroid tumors can metastasize to the contralateral axillary lymph nodes, and in such cases it is important to make a differential diagnosis with a breast cancer lymph node metastasis [27]. Furthermore, extended lymph node dissections in patients with thyroid cancer may be more challenging and high-risk than simple thyroidectomies; however, there is no evidence in the literature that they can significantly increase the complication rates [28–33].

Our decision to evaluate the length and the extensibility of the neck as predictive factors of difficulty was empiric and according with most of the neck surgeons that consider fundamental a satisfactory exposure of the surgical site. This can be achieved with hyperextension of the neck and eventually interposition of a support between the table and patient’s shoulder blades. Reasonably, in patients with short or inflexible neck the surgeon may experience greater difficulty during surgery because of a restricted operating field.

Kwak et al. [18] in 2017 suggested a score, graded after surgery, identifying the duration of the operation as a factor of surgical difficulty in thyroidectomy. Although they demonstrated that the young age and the male sex are predictive factors of difficult thyroidectomy in terms of operative times, the incidence of complications is not statistically higher in the group of potentially difficult thyroidectomies. The results are comparable to those of previous studies reporting that a higher BMI could be considered a predictive factor of longer operating times and higher morbidity [34]; other Authors suggested that neck circumference, instead of BMI, might be correlated with the duration of the operation as a predictor of difficulty [19].

In 2014 Schneider et al. [5] proposed a Thyroidectomy Difficulty Scale (TDS) based on postoperative evaluation of four parameters (vascularization, friability, fibrosis and gland size) in order to predict the surgical difficulty. After analysis of the data, they observed that increased vascularization of the thyroid gland is more correlated to hyperthyroidism, similarly with the presence of thyroiditis that influences the development of parenchymal fibrosis. The Authors concluded that high scores were correlated with longer operative times and higher risk of complications.

Subsequently, Mok et al. [17] compared the variables used for the TDS with the pathological features of the population and underlined that patients with hyperthyroidism, high levels ​​of thyroglobulin and positivity for anti-thyroglobulin antibodies showed a higher overall score, therefore, were subjects of major risk of complications.

In the present study, the incidence of complications was negligible with quite homogeneous distribution among the three groups of patients. No permanent hypoparathyroidism or RLN injury have been reported. Thus, we can argue that the predictive factors of difficulty in thyroidectomy identified by our score did not affect the rate of complications, contrarily to previous studies [5, 17]. Even analysis of the data regarding the length of hospitalization did not show statistical correlation with our score (Table 4).

However, the results related to the operative times were different; there is strong evidence that a higher score level correlates with longer duration of the operation and therefore with an increased complexity of the surgical procedure (Table 4). Nevertheless, no association was shown in our series between the rate of perioperative complications and the complexity or duration of the operation. We strongly believe that total thyroidectomy performed by experienced surgeons with microsurgical technique under optical magnification permits to significantly reduce the incidence of permanent hypoparathyroidism and RLN lesions, as already demonstrated in our previous studies and confirmed by numerous evidences in the literature [35–40].

The current study has some limitations. Firstly, the subjective criteria in the definition of preoperative parameters as risk factors and secondly the empirically estimated threshold values, or even the absence of intermediate values in the categorical variables suggested for the score calculation. However, this has been done intentionally in order to make the score more intuitive and, therefore, easier to calculate. Finally, the number of patients who composed our study group from a single institution is certainly too low to obtain universally consolidated results.

Despite the limitations, we must recognize some strengths of this study; it is a prospective study, based on data collected directly during the clinical assessment of each patient and not through the medical records. Our thyroidectomy difficulty score is the only one existing in the literature than was formulated exclusively in accordance with preoperative parameters. The intention was to provide the surgeons with a valid tool to predict difficulty in thyroidectomy and to identify the patients at risk of complications. Moreover, this information could be useful to improve the informed consent counseling and the operating theatre planning. Finally, the parameters considered as predictors of difficulty, were heterogeneous and therefore more complete: some concerned the demographic characteristics of the patient, others their anatomo-pathological features, others the characteristics of the thyroid gland.

Further studies, with a larger cohort of patients, are needed to validate our results and to formulate a universally-accepted preoperative predictive score of difficulty in thyroid surgery.

## Conclusions

The comparison among the groups of patients who underwent total extracapsular thyroidectomy with microsurgical technique, defined preoperatively by the score as low, medium and high risk of complications, allowed us to make some interesting considerations regarding the incidence of complication, such as RLN injuries and hypoparathyroidism, either transient or permanent.

Our data revealed that these complications, besides negligible and always transitory, are distributed quite homogeneously among the subgroups of patients, so we can conclude that the predictors of difficulty in thyroidectomy are not correlated with morbidity rates, as contrary suggested by previous studies, but only with operative times, which were significantly increased in patients with higher score.

Although our results have limited statistical significance, they support our belief in the fundamental role of the microsurgical technique in thyroid surgery, as a valuable instrument to recognize and overcome any foreseeable or unexpected difficulties and to maintain the percentage of complications insignificant.

In addition, the prediction of a difficult thyroidectomy may be helpful for less experienced surgeons to increase the level of attention, especially in complex situations.

The results of our study could be useful not only to predict the difficulty of a total thyroidectomy but also to optimize the schedule of the operating room, reduce the costs and improve the management of the patients and the available resources.

## Abbreviations

BMI: body mass index
RLN: recurrent laryngeal nerve
TDS: thyroidectomy difficulty scale
